# Characterization, Stability and Biological Activity In Vitro of Cathelicidin-BF-30 Loaded 4-Arm Star-Shaped PEG-PLGA Microspheres

**DOI:** 10.3390/molecules23020497

**Published:** 2018-02-23

**Authors:** Yueli Bao, Shanrong Wang, Hongli Li, Yunjiao Wang, Haiyun Chen, Minglong Yuan

**Affiliations:** 1 Engineering Research Center of Biopolymer Functional Materials of Yunnan, Yunnan Minzu University, Kunming 650500, China; Baoyueli1993@163.com (Y.B.); honglili_1982@163.com (H.L.); YunjiaoWang@yeah.net (Y.W.); 2 Yunnan Rural Leader College, Yunnan Agricultural University, Heilongtan, Kunming 650201, China; ndxb86@126.com

**Keywords:** cathelicidin-BF-30, 4-arm-PEG-PLGA, microspheres

## Abstract

BF-30 is a single chain polypeptide of an N-segment with an α-helix from cathelicidin gene encoding, and it contains 30 amino acid residues, with a relative molecular mass and isoelectric point of 3637.54 and 11.79, respectively. Cathelicidin-BF-30 was entrapped in four-arm star-shaped poly(ethylene glycol-b-dl-lactic acid-co-glycolic acid) block copolymers (4-arm-PEG-PLGA) by a double-emulsion solvent-evaporation method. Three release phases of cathelicidin-BF-30loaded 4-arm-PEG-PLGA microspheres were observed, including an initial burst-release phase, followed by a lag phase with minimal drug release and finally a secondary zero-order release phase. The delivery system released BF-30 over more than 15 days in vitro. Furthermore, the material for preparing the microspheres has good biocompatibility and biodegradability. Additionally, based on the drug resistance of food pathogenic bacteria, the antibacterial effects of BF-30 on *Shigella dysenteriae CMCC 51105* (*Sh. dysenteriae CMCC 51105*), *Salmonella typhi* (*S. typhi*) and *Staphylococcus aureus* (*S. aureus*) as well as the stability of the in vitro release of the BF-30-loded microspheres were studied. The α-helix secondary structure and antibacterial activity of released BF-30 were retained and compared with native peptide. These BF-30 loaded microspheres presented <10% hemolysis and no toxicity for HEK293T cells even at the highest tested concentration (150 μg/mL), indicating that they are hemocompatible and a promising delivery and protection system for BF-30 peptide.

## 1. Introduction

In recent years, antibacterial peptides (AMPs) have been extensively studied and are considered the new generation of antibiotics. Antimicrobial peptides play an important role in the innate immune system. The biggest advantage of antimicrobial peptides is that they do not cause bacterial resistance [1,2,3]. The main shortcoming of the peptides is their short half-lives in serum [4]. The AMPs are small, gene-encoded peptides that show a broad range of activity against Gram-negative as well as Gram-positive bacteria, fungi, and mycobacteria (Zasloff, 2002) [5]. Currently, approximately 2000 different sources of antimicrobial peptides have been discovered [6,7]. Cathelicidin-BF (BF-30) is a cathelicidin-like antimicrobial peptide of 30 amino acids from non-mammalian vertebrates encoded by the gene of *Bungarus fasciatus*. The secondary structure of BF-30is an amphipathic α-helical conformation and is positive [8]. The results showed that BF-30 exhibited broad-spectrum antibacterial activity and was not susceptible to drug resistance compared with antibiotics [8]. Cathelicidin-BF (BF-30) is a lysine- and phenylalanine-rich antimicrobial peptide, and it exhibits broad antimicrobial activity against bacteria and fungi with the amino acid sequence KFFRKLKKSVKKRAKEFFKKPRVIGVSIPF [9]. Its antimicrobial activity in vivo and antimicrobial mechanism through interference with the cytoplasmic membrane integrity has been recently revealed [8,10]. It has been found that BF-30 can effectively reduce the infection of *Pseudomonas aeruginosa* in the lungs and liver of burned mice and prevent subsequent systemic infection and inflammation [8]. Previous work has demonstrated that BF-30 has medicinal potential.

Nowadays, standards of living have improved, and food safety has become a focus of attention. Naturally, diseases caused by food-borne pathogens have also received widespread attention. Food-borne diseases such as *Listeria* and *Salmonella* can cause many serious infections due to food contaminated by pathogenic bacteria or their toxins [11]. *Salmonella* is a major cause of food-borne disease outbreaks and infections in many countries. Due to the high prevalence of *Salmonella* in livestock, animal-derived foods such as milk and poultry are often associated with human infection [11,12]. However, more than 10 types of iatrogenic or food-borne microorganisms, such as *Enterococcus*, *Salmonella* and *S. aureus*, have produced a wide range of antimicrobial resistance, which poses a serious threat to the health of human beings worldwide [13].

The polymeric material poly(d,l-lactic acid-co-glycolic acid) (PLGA) has been used as a Food and Drug Administration (FDA) [14] approved drug carrier for the encapsulation of many drugs, such as small molecules, peptides, proteins and so on [15,16]. In recent years, PLGA has been used as a carrier for risperidone, exenatide, a novel antimicrobial peptide GIBIMP5S9K and many other drugs [17,18,19]. PLGA has good biocompatibility and biodegradeability [17,20]. Therefore, the antimicrobial peptide BF-30 was embedded into PLGA by a double-emulsion solvent-evaporation method to prepare microspheres in order to solve the problem of the short half-life of antimicrobial peptide BF-30, and the stability and release behavior of BF-30 loaded microspheres should be further studied. However, the encapsulation efficiency of hydrophilic drugs in hydrophobic polymers (such as PLGA) microspheres is not high because the compatibility of this drug with polymers is very low [21]. Moreover, the temperature-sensitive sol–gel transition behavior of biodegradable 4-arm star-shaped PEG-PLGA block copolymer aqueous solution has been investigated, and research indicates the possibility of 4-arm-PEG-PLGA block copolymers as an injectable drug-delivery carrier [22]. Therefore, it was proposed to enhance the hydrophilicity of PLGA, and we chose 4-arm-PEG-PLGA to investigate in this study. The entrapment efficiency of cathelicidin-BF-30-4-arm-PEG-PLGA microspheres was high as well as stable, and sustained release could be achieved by optimizing the conditions of the preparation of microspheres. The aim of this study was to obtain stable and sustained release of loaded antimicrobial peptide BF-30 microspheres between two and three weeks.

## 2. Results and Discussion

### 2.1. Characterization of 4-Arm-PEG-PLGA

**Figure 1:**
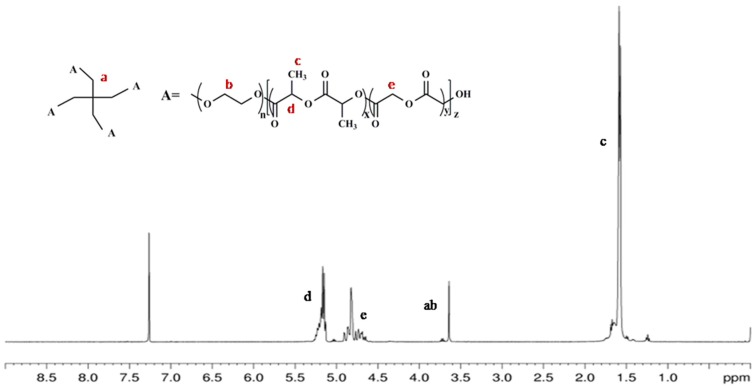
^1^H nuclear magnetic resonance (NMR) spectrum of 4-arm-PEG-PLGA.

The successful synthesis of 4-arm-PEG-PLGA was determined mainly by the appearance of the peaks of δ = 1.58 ppm (c, –O–CH(CH_3_)–COO), δ = 4.8 ppm (e, –O–CH_2_–COO), δ = 5.2 ppm (*d*, –O–CH(CH_3_)–COO–), and δ = 3.6 ppm (a,b,4-arm-PEG) in the ^1^H nuclear magnetic resonance (NMR) spectrum in Figure 1. ^1^H-NMR spectra were recorded with a Bruker 400 MHz, and deuterated chloroform was used for detection.

### 2.2. Scanning Electron Microscopy (SEM) Analysis and Size Distribution of Microspheres

**Figure 2:**
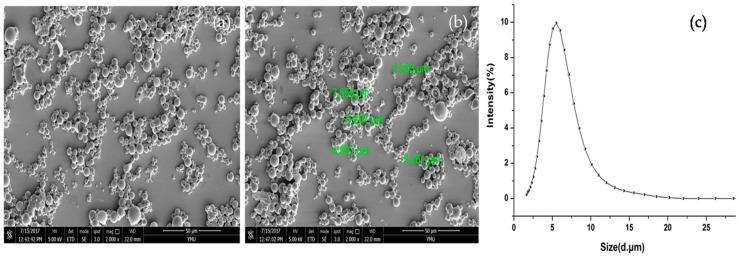
Scanning electron microscopy (SEM)images (**a**,**b**) and size distribution (**c**) of 4-arm-PEG-PLGA microspheres.

Representative scanning electron microscopy (SEM) micrographs and the size distribution for the 4-arm-PEG-PLGA microspheres used in this study are shown in Figure 2. The SEM images showed that the microspheres were spherical and had a smooth surface. However, some of the microspheres were adhesive, and the sizes of these microspheres differed. Uneven size is a defect of microspheres prepared by the double-emulsion method.

As reported, the exenatide-loaded PLGA microspheres were prepared by a SPG premix membrane emulsification technique combined with a double emulsion (W_1_/O/W_2_) solvent evaporation method [18]. The drug exenatide and BF-30 are both peptides, and they have similar properties. Therefore, the BF-30-loaded 4-arm-PEG-PLGA microspheres were compared with the exenatide-loaded PLGA microspheres, and we found that the size of the 4-arm-PEG-PLGA microspheres was smaller than that of the PLGA microspheres by examining the particle size distribution. The GIBIMP5S9K peptide-loaded PLGA nanoparticles were extremely sticky, and the dispersion was worse than the BF-30-loaded 4-arm-PEG-PLGA microspheres, as seen by the SEM images, which were compared to the new antimicrobial GIBIMP5S9K peptide-loaded PLGA nanoparticles [19].

### 2.3. Drug Loading

As the PEG segments and PLGA segments have different solubilities, this tends to induce micro-phase separation, which occurs locally. Consequently, the micelle core is spontaneously formed. First of all, the compatibility between the core chains and drug molecules is of the utmost importance for physical drug loading. Hence, the favorable factors for compatibility are essentially better for increasing the drug loading [23]. In this investigation, the drug molecular weight is high and the water solubility of the drug is very good. Therefore, it is hard to obtain a high drug loading.

Drug loading is characterized by the BF-30 encapsulation efficiency (EE), BF-30 loading content (LC), and BF-30 loading efficiency (LE). It is generally true that as long as the drug loading rate is higher, the entrapment efficiency is higher, and high encapsulation efficiency is important for the drug release of microspheres. In other words, a higher LE can meet the demands for a longer-term release or lower injection dosage of the microspheres. BF-30-loaded 4-arm-PEG-PLGA microspheres were prepared using a double-emulsion solvent-evaporation method. The concentration of BF-30 was determined by high-performance liquid chromatography (HPLC). In this study, the EE and LE values of the BF-30-loaded 4-arm-PEG-PLGA microspheres are 74.66 ± 3.381% and 2.873 ± 0.1762%, respectively.

### 2.4. In Vitro Degradation of 4-Arm-PEG-PLGA Microspheres

**Figure 3:**
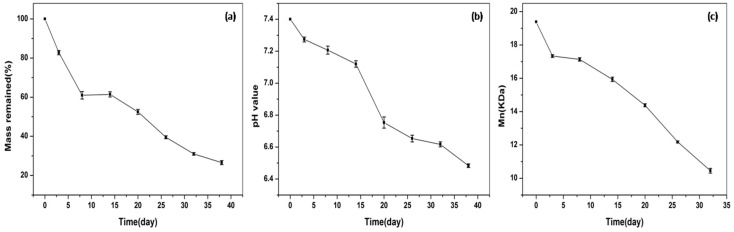
The degradation profiles of microspheres in phosphate-buffered saline (PBS) (pH = 7.40): the mass (**a**) pH (**b**) and Mn (**c**) changed with time.

**Figure 4:**
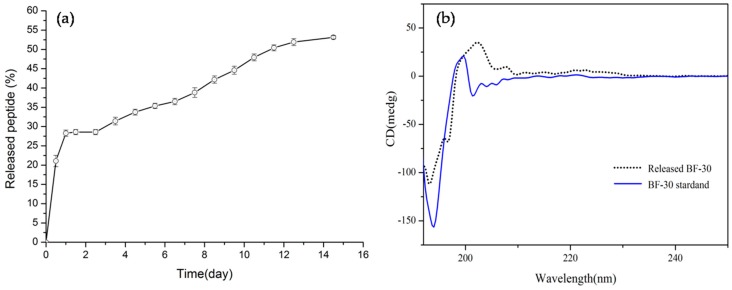
Release of BF-30 loaded microspheres in vitro for two weeks (**a**). Circular dichroism spectra of native and released BF-30 in one dayof release (**b**).

Degradation of the diblock copolymers depends on several factors, such as the type of chemical bonds, mobility of water within the polymer, and pH of the polymer solution [14]. Moreover, an accelerated molecular weight loss was observed by adding the PEG block in diblock copolymer, indicating that the incorporation of the PEG segment as a hydrophilic part can change the physicochemical properties of hydrophobic and biodegradable PLGA block segments. Accordingly, the addition of the 4-arm-PEG segment as a hydrophilic part into the hydrophobic PLGA led to faster erosion of the PLGA part due to the better accessibility of water to the ester bonds of the PLGA block. In our research, 4-arm-PEG-PLGA microspheres showed continuous weight loss over time for more than 30 days and a decrease in the average molecular weight (Figure 3). Furthermore, the cleavage of the PLGA block could induce a pH change in the medium. Hence, the variation of the pH in phosphate-buffered saline (PBS) as the medium was also measured to estimate the degradation of PLGA in the microspheres. The degradation medium showed the decline in pH over 30 days, as shown in Figure 4. In this study, 50 mg of blank 4-arm-PEG-PLGA microspheres and 10 mL PBS (pH = 7.40, 10 mM, containing 0.02% NaN_3_ and 0.9% NaCl) were placed into a 15 mL centrifugal tube.

### 2.5. In Vitro Release of BF-30-Loaded Microspheres

The characteristics of drug release are also related to the water and degradation behavior of copolymers. Commonly, burst release occurs during the initial phase because of the interfacial drug loading [23,24]. It was shown previously that a higher drug loading resulted in slower drug release [23]. The rapid hydration due to PEG leads to a swollen matrix, which released the protein in a slow and continuous way. Additionally, the cumulative release of the drug decreased with an increasing number of arms in the st-PLGA–PEG copolymers, which is probably because the st-PLGA–PEG copolymer with many arms forms a more compact core [25]. Finally, the different release kinetics of the microspheres depended on the copolymer degradation, with the lowest molecular weight microspheres showing a higher release rate due to faster degradation and a looser structure.

Drug release from microspheres is normally governed by a combination of polymer erosion and drug diffusion. In general, drug-loaded microspheres have three release phases that can be observed, including an initial burst release phase, a lag-phase, and finally a release plateau [17,19,26]. We investigated the BF-30-loaded 4-arm-PEG-PLGA microspheres, which were prepared by the double-emulsion solvent-evaporation method. As shown in Figure 4a, BF-30-loaded microspheres had an initial burst-release phase in 12 h, and the release percentage reached 21.07 ± 1.47%. This was followed by a lag phase with minimal drug release and a secondary zero-order release phase, with the BF-30 released at approximately two weeks greater than 50%.

### 2.6. Circular Dichroism Spectral (CD) Analysis

Like most cathelicidins, the secondary structure of BF-30 was investigated by circular dichroism [9,10,27]. In the far ultraviolet circular dichroism spectra (far-UV CD) of the native and released BF-30, the trends of the spectra of the two samples are approximately the same, as shown in Figure 4b. The released BF-30 shows no difference in secondary structure compared to the control, however, there is little evidence for the adoption of any helical or other regular secondary structure conformation. In addition, a slightly different extent of secondary structure of the released BF-30 and the native BF-30 was visible in the CD spectrum. This finding suggests that the released protein was affected by the released medium, which included the degradation substances of the BF-30-loaded 4-arm-PEG-PLGA microspheres and a change in pH. Previous literature reported that the secondary structure of the peptide is an α-helical type [8,9,10,27]. However, according to the CD spectrum, the content of the helical conformation cannot be revealed. This result could be caused by the longer storage time of samples, the instability of the peptide and instrument noise, as well as the changes in the medium environment of the released peptide (for instance, the change of pH will destroy ionic bonds that stabilize the helical structure). CD spectra were measured at the concentration of the sample in PBS (pH = 7.4).

### 2.7. In Vitro Cytotoxicity

To investigate the cytotoxicity of the BF-30 loaded 4-arm-PEG-PLGA microspheres, human HaCaT keratinocyte cells and human HEK293T embryonic kidney cells were chosen to be incubated with various concentrations of the as-prepared microspheres. HaCaT cells are utilized for their high capacity to differentiate and proliferate in vitro [28]. Cathelicidin-BF significantly inhibited pro-inflammatory factors’ secretion in human monocytic cells and P. acnes-induced O_2_^−^ production of human HaCaT keratinocyte cells [29]. The HEK293T cell line is easy to raise and of fast proliferation. HEK293T cells were used to examine the cytotoxicity of peptide ZY13, which were designed based on cathelicidin-BF [30].

**Figure 5:**
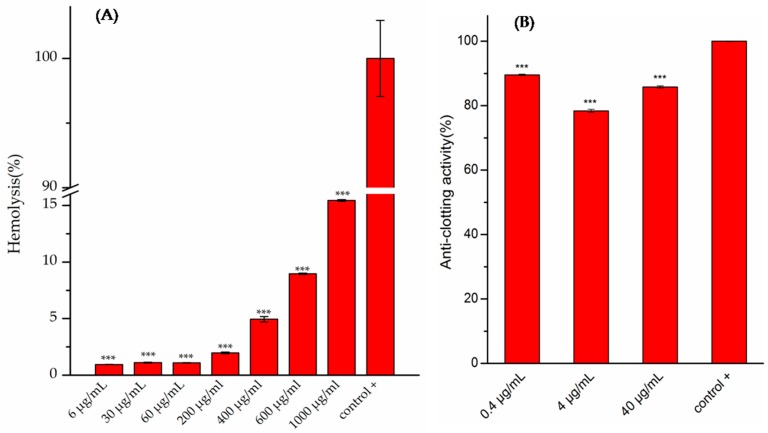
Hemolytic activity (**A**) and anti-clotting activity (**B**) of BF-30 encapsulated in 4-arm-PEG-PLGA microspheres. *** *p* < 0.001 versus positive control.

**Figure 6:**
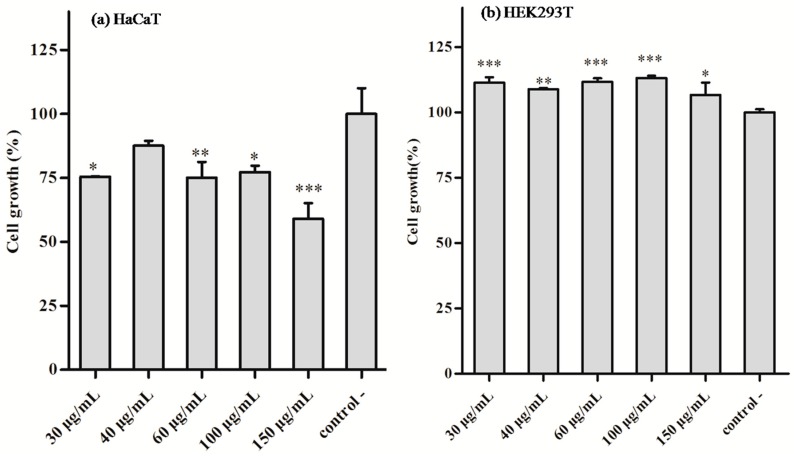
The different concentrations of BF-30-loaded microspheres were used to determine the cytotoxicity. The cell growth of 4-arm-PEG-PLGA microspheres loaded with BF-30 using human HaCaT keratinocyte cells (**a**) and human HEK293T embryonic kidney cells (**b**). * *p* < 0.05, ** *p* < 0.01 and *** *p* < 0.001 versus microspheres without compound (negative control). Values are expressed as the mean ± SD of three independent experiments.

HEK293T and HaCaT cells were cultured in 96-well plates, as described above. The results of the CCK8 assay are shown in Figure 5, using PBS as the negative control. The BF-30-loaded microspheres showed a >75% cell growth below the concentration (100 μg/mL), which was used to evaluate the cytotoxicity of the HaCaT cells, as shown in Figure 6a. It can be seen that the BF-30-loaded microspheres are cytotoxic to HaCaT cells at the concentration of 100 μg/mL as well as lower microsphere concentrations. As shown in Figure 6b, the cytotoxicity of the BF-30-loaded microspheres cultured with HEK293T cells was determined after 48 h. The data show that the cell-growth values with different tested concentrations are higher than the negative control group. In other words, the cell vitalities of HEK293T cells are greater than without microspheres. Accordingly, the relative cell vitalities of HEK293T cells incubated with all of the BF-30-loaded microspheres after 48 h were greater than 100%, which indicated that no toxicity occurred at the concentrations tested, even at the highest tested concentration (150 μg/mL). Moreover, the BF-30-loaded microspheres were non-toxic to the HEK293Tcells and suitable as an extracellular matrix for cell growth, however, the same concentration BF-30-loaded microspheres inhibit proliferation of HaCaT cells. This is probably associated with the characteristics of two kinds of cells. HEK293T cells derive from HEK293 cells. The article reported that no significant cytotoxicity against human HEK293 embryonic kidney cells was observed after treatment by peptide ZY13 at various concentrations from 0.39 μg/mL to 100 μg/mL [30]. HaCaT cells are non-tumor derived from human normal-skin immortalized keratinocytes, similar to the differentiation characteristics of normal human keratinocytes, which can reproduce more than 150 generations, but are not tumor cells. The absorbance of the resulting solution at 450 nm was measured. The experiments were performed in triplicate.

### 2.8. Hemolytic Activity and Anti-Clotting Analysis

Rabbit red blood cells were used to check for the hemolytic capability in our experiments. As shown in Figure 5A, BF-30-loaded 4-arm-PEG-PLGA microspheres had low hemolytic activity against the red blood cells of rabbits, and at the highest tested concentration of 1000 μg/mL the hemolytic activity was approximately 15%. As reported, BF-30 had a relatively low hemolytic activity against the erythrocytes of sheep, even at the highest tested concentration of 320 μg/mL [31]. Therefore, free peptide and 4-arm-PEG-PLGA microspheres loaded with peptide had low hemolytic activities. In general, 10% and 25% hemolysis are the relative boundaries. In previous studies, these peptide-loaded nanoparticles could be considered hemocompatible substances, which could potentially be administered parenterally because a compoundcausing <10% hemolysis is considered non-hemolytic and one causing >25% hemolysis is classified as hemolytic [19,32,33].

Three solutions with different concentrations of BF-30-loaded microspheres were chosen to measure the anti-clotting activity as shown in Figure 5B. The minimum of the anti-clotting activity is 78.37 ± 0.4924%, and the maximum is 89.55 ± 0.2141%. The data showed that the anti-clotting activity of BF-30-loaded microspheres is acceptable.

### 2.9. Antibacterial Activity of BF-30 Free and Peptide-Loaded Microspheres

BF-30 possessed strong and rapid bactericidal activity against *Escherichia coli* (*E. coli*) and *S. aureus* but only exhibited bacteriostatic activity against certain drug-resistant bacteria [30]. In our research, the antimicrobial activities of cathelicidin-BF-30 against *Sh. dysenteriae CMCC 51105*, *S. typhi* and *S. aureus* were determined by the bacteriostatic zone method [30,34]. There is a bacteriostatic zone around the filter paper on the plate, which is coated with bacteria. This indicates that the drug has antibacterial activity, and the size of the bacteriostatic zone can be used to evaluate the antimicrobial activity.

**Figure 7:**
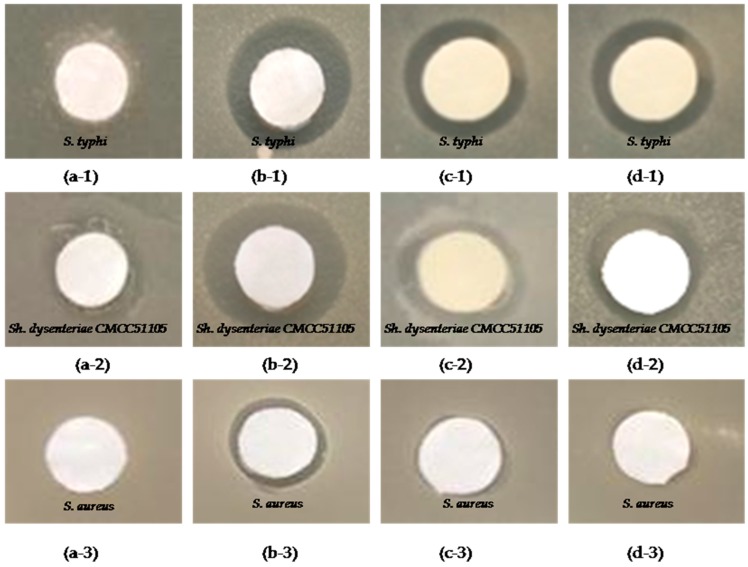
The antimicrobial activities of free BF-30 (**b**), extracted BF-30 (**c**), and released BF-30 (**d**). PBS (**a**) was used as a negative control in this investigation.

In this investigation, free BF-30, BF-30 extracted from 4-arm-PEG-PLGA microspheres, and BF-30 released from 4-arm-PEG-PLGA microspheres were chosen to observe the antimicrobial activities against the three kinds of bacteria. These bacteria were cultured with the tested samples in the culture dish. All of the culture dishes were cultured for 20 h at a 37 °C constant temperature to observe whether there was inhibition-zone formation. The results of the bacteriostatic effect on these drug-resistant bacteria are shown in Figure 7. First, it can be seen from the picture that the bacteriostatic effects of the BF-30 on *S. typhi* and *Sh. dysenteriae CMCC 51105* are better than *S. aureus*. Second, the bacteriostatic effect of the free BF-30 on these bacteria is better than those of the BF-30 extracted from 4-arm-PEG-PLGA microspheres and the BF-30 released from 4-arm-PEG-PLGA microspheres, which is due to the fact that the concentrations of the extracted BF-30 and the released BF-30 were lower than that of the free BF-30. Finally, the group in (a) did not form an inhibition-zone, which was without peptide but with PBS as a negative control.

## 3. Materials and Methods

### 3.1. Materials

4-Arm-PEG-OH (Mw = 10,000 Da) was purchased from the SeeBio Incorporated Company (Shanghai, China). 4-arm-PEG-PLGA was prepared through condensation polymerization in our lab. Polyvinyl acetate (PVA, Mw = 75,000 Da, 88% alcoholysis, biochemical reagent) was purchased from the Shanghai Jingchun Reagent Company Limited (Shanghai, China). Dichloromethane, ethyl alcohol and isopropanol (analytical grade) were purchased from the Tianjin Damao Chemical Reagent Factory (Tianjin, China). Disodium hydrogen phosphate, monopotassium phosphate, trifluoroacetic acid, sodium azide, and sodium chloride (analytical grade) were purchased from the Chengdu Kelon Reagent Company (Chengdu, China). Acetonitrile (HPLC grade) was from MREDA Technology Inc. (Beijing, China). BF-30 with a Mw of 3637.50 Da was purchased from the China Peptides Co. Ltd. (Shanghai, China). CCK8 was purchased from MedChem Express (MCE). The cells were purchased from the Kunming cell bank, a typical culture preservation of the Committee of the Chinese Academy of Sciences. A male rabbit (2.1 kg) was obtained from the Laboratory Animal Unit of Kunming Medical University (Kunming, China).

### 3.2. Preparation of 4-Arm-PEG-PLGA

**Scheme 1:**
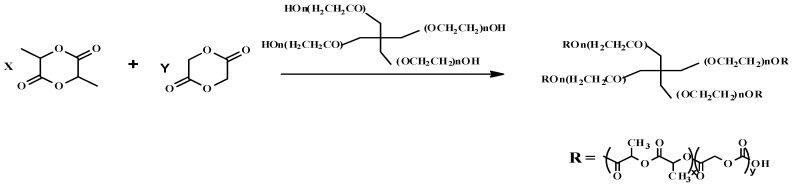
Synthesis of 4-arm-PEG-PLGA.

PEG and PLGA have already been approved by the FDA, and PEG-PLGA copolymers are nontoxic after hydrolysis. The applications of PEG–PLGA polymeric micelles have been the subject of several studies [35,36,37,38,39]. The 4-arm-PEG-PLGA copolymer was synthesized by ring-opening polymerization, and it was prepared via a three-step synthetic strategy. First, the lactide (d,l-LA) monomer was synthesized by lactic acid. Second, the glycolide (GA) monomer was synthesized by glycolic acid. Finally, the 4-arm-PEG-PLGA copolymer was successfully synthesized via the ring-opening polymerization of d,l-LA and GA with a catalytic amount of Sn(Oct)_2_. The method of synthesizing 4-arm-PEG-PLGA refers to the literature [22]. As shown in Scheme 1, the synthetic procedure of 4-arm-PEG-PLGA was as follows: d,l-lactide (0.083 mol), glycolide (0.033 mol), 4-arm-PEG (0.317 g, 2% of the total mass of LA and GA) and Sn(Oct)_2_ (1‰ of the monomer) were added into a round-bottom flask.

### 3.3. Preparation of 4-Arm-PEG-PLGA Microspheres Loaded with Cathelicidin-BF-30

**Figure 8:**
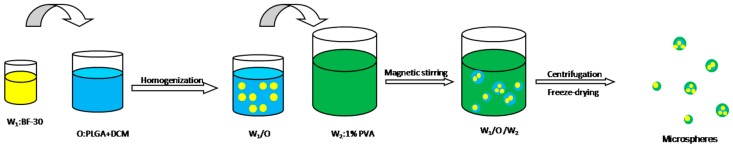
Preparation process of microspheres using the emulsification technique.

The double emulsion (W/O/W) is a classical method for loading water-soluble drugs [40,41,42]. A water/oil/water (W_1_/O/W_2_) double-emulsion solvent-evaporation method was employed to prepare microspheres containing antimicrobial peptide BF-30 and blank microspheres (Figure 8). Briefly, 15 mg of BF-30 was dissolved in 0.25 mL of water, and 250 μL of BF-30 aqueous solution was mixed with 5 mL of dichloromethane containing 4-arm-PEG-PLGA in an ice-bath under high-speed homogenization for a minute and a half with Span80 as a surfactant. The resulting first emulsion was slowly added to 10 mL of PVA (1%) under agitation, and emulsification continued at 21,000 rpm for 3 min. The emulsion was added into 250 mL of water with 10 mL of isopropyl alcohol and stirred continuously for 4 h at room temperature and atmospheric pressure until complete solvent evaporation. After the microspheres had formed, they were collected by centrifugation at 65,000 rpm for 9 min, rinsed with water three times, resuspended with 1 mL of 0.5% mannitol, freeze-dried for 20 h and stored at −20 °C. Blank microspheres were prepared by the same method.

### 3.4. Observation of the Surface Morphology and Size-Distribution Measurements

The shape and surface morphology of 4-arm-PEG-PLGA microspheres were observed by a NOVA NANOSEM-450 (FEI, Hillsboro, OR, USA) scanning electron microscope (SEM). The particle size distribution was measured by laser diffraction using a Mastersizer S3500 (Microtrac, Shanghai, China).

### 3.5. Determination of the BF-30 Content in 4-Arm-PEG-PLGA Microspheres

A high encapsulation efficiency of microspheres is a key index of an excellent microspheres-delivery system. Twenty milligrams of microspheres were dissolved in 800 μL of dichloromethane and transferred to a small liquid funnel, and then, 600 μL of ethyl alcohol, 800 μL of PBS, and 500 μL of acetonitrile were added. The concentration of peptide in the supernatant liquid was measured by HPLC and compared with the standard curve data of known concentrations of BF-30 solutions. Next, the supernatant liquid was filtered and then injected into a reversed-phase HPLC (RP-HPLC, Agilent Technologies 1200 Series, Agilent Technologies, Palo Alto , CA, USA) [18,19] system to determine the concentration of BF-30. The determination was performed at room temperature using a Waters C18 (Symmetry Shield™, RP18, 5 μm, 4.6 × 250 mm column, Waters, Milford, MA, USA) chromatographic column. A linear gradient elution was performed from 20% to 75% acetonitrile trifluoroacetic acid solution (0.1%) (mobile phase A) for 15 min, with ultrapure water containing 0.1% TFA as mobile phase B. The flow rate was 1 mL/min, and the UV absorbance was determined at 220 nm [43,44]. Drug loading is characterized by the BF-30 encapsulation efficiency (EE), BF-30 loading content (LC), and BF-30 loading efficiency (LE) defined as follows:

(1) $$\mathrm{LC}=\frac{\mathrm{weight}\;\mathrm{of}\;\mathrm{BF}–30\;\mathrm{in}\;\mathrm{microspheres}}{\mathrm{total}\;\mathrm{weight}\;\mathrm{of}\;\mathrm{microspheres}}$$

(2) $$\mathrm{LE}=\frac{\mathrm{weight}\;\mathrm{of}\;\mathrm{BF}–30\;\mathrm{in}\;\mathrm{microspheres}}{\mathrm{initial}\;\mathrm{weight}\;\mathrm{of}\;\mathrm{BF}–30}$$

(3) $$\mathrm{EE}=\frac{\mathrm{weight}\;\mathrm{fraction}\;\mathrm{BF}–30\;\mathrm{in}\;\mathrm{microspheres}}{\mathrm{initial}\;\mathrm{weight}\;\mathrm{fraction}\;\mathrm{of}\;\mathrm{BF}–30}$$

### 3.6. In Vitro Release Study

Approximately 100 mg of BF-30-loaded 4-arm-PEG-PLGA microspheres was incubated in a capped centrifugal tube with 5 mL of 10 mM PBS medium (pH = 7.4) under agitation at 37 °C placed in a constant temperature oscillator. The tubes were removed and the samples were centrifuged at 5000 rpm for 5 min at predetermined intervals. The supernatants were collected periodically and replaced with fresh buffer of equal volume. The concentration of BF-30 in the supernatant was determined by HPLC. All of the release experiments were performed in triplicate.

### 3.7. In Vitro Biodegradation Study

The in vitro biodegradability of blank 4-arm-PEG-PLGA microspheres was evaluated by measuring the changes in molecular weight at 37 °C in PBS (pH = 7.4) with shaking at 120 rpm. The samples were centrifuged at specific time intervals and the degraded microspheres were washed with distilled water to rinse off the buffer salt. The remaining microspheres were freeze-dried and their molecular weight changes were determined by gel permeation chromatography (GPC). In addition, the pH changes of upper solutions were measured with a pH meter (PH-3C, China).

### 3.8. Analysis of the Secondary Structure of BF-30 Released from Microspheres

Far-UV CD is an effective method for detecting the secondary structure of a protein or polypeptide. Released BF-30 was measured and compared with the native peptide. The solution of released peptide was analyzed by CD, and the optical path of the quartz sample cell was 0.5 cm in the far ultraviolet region (190–270 nm) with a 1 nm bandwidth and determination at room temperature. Meanwhile, PBS, which was the release medium, was used as a blank. 

### 3.9. In Vitro Cytotoxicity Analysis

The cytotoxicity of BF-30-loaded 4-arm-PEG-PLGA microspheres to HaCaT cells was evaluated using the CCK8 assay [45,46]. These cells were cultured in RPMI-1640 medium containing 10% fetal calf serum. The density of the growth of cells reached 80–90%, and the cells were selected to plate in 96-well plates. The adhered cells were cultured with low serum or serum-free medium for more than 16 h. These cells were cultured with different concentrations of sample for 48 h, and every concentration was evaluated in three wells. Then, 20 μL of CCK8 was added to the wells. After incubation at 37 °C for 3 h, the absorbance at 450 nm was then measured using an enzyme-linked immunosorbent assay (ELISA) reader. The cell growth, indicating the magnitude of toxicity, was calculated according to the following equation:

(4) $$\mathrm{cell}\;\mathrm{growth}\left(\%\right)=\frac{{\mathrm{Abs}}_{\mathrm{sample}}}{{\mathrm{Abs}}_{\mathrm{negative}\;\mathrm{control}}}\times 100\%$$

### 3.10. In Vitro Hemolysis Test

A hemolysis assay was undertaken using rabbit red blood cells in liquid medium, as reported previously [32]. Serial dilutions of the testing sample were used, and after incubation at 37 °C for 30 min, the cells were centrifuged, followed by measurement of the absorbance of the supernatant at 540 nm [31]. Pure water with rabbit red blood cells was used as the positive control, and saline with rabbit red blood cells was used as the negative control in this investigation. All of the samples were evaluated in triplicate. The hemolytic activity is defined as follows:

(5) $$\mathrm{Hemolysis}\left(\%\right)=\frac{{\mathrm{Abs}}_{\mathrm{sample}}-{\mathrm{Abs}}_{\mathrm{negative}\;\mathrm{control}}}{{\mathrm{Abs}}_{\mathrm{positive}\;\mathrm{control}}-{\mathrm{Abs}}_{\mathrm{negative}\;\mathrm{control}}}\times 100\%$$

### 3.11. In Vitro Anti-Clotting Test

The sample was dissolved in distilled water and mixed with 24.52 μg/mL, 245.2 μg/mL and 2.452 mg/mL solutions, respectively. The final concentrations were 0.4 μg/mL, 4 μg/mL and 40 μg/mL. Two hundred microliters of solution was placed in a 15 mL centrifuge tube and kept at constant temperature for 5 min at 37 °C. Then, 50 μL of fresh anticoagulant rabbit blood was injected into the samples and kept for 5 min at constant temperature. Next, 10 μL of calcium chloride solution (0.2 mL/L) was added into the centrifuge tube with the sample, and the centrifuge tube was shaken to mix the calcium chloride with blood, followed by resting at a constant temperature for 5 min. The sample was taken out of the centrifuge tube, and 12 mL of deionized water was added. Then, the absorbance of the supernatant was measured at a wavelength of 540 nm by UV spectrophotometry. Twelve milliliters of deionized water with 50 μL of whole blood was used as the control. All of the samples were determined five times to obtain the average value. The anti-clotting activity is defined as follows:

(6) $$\mathrm{BCI}=\frac{I_{0}}{I_{w}}\times 100\%$$

where I_0_ was the relative absorbance of the mixture of blood and calcium chloride after contact with the sample at a set time and I_w_ was the relative absorbance of the mixture of blood with a certain amount of deionized water.

### 3.12. Antimicrobial Activity of the Released Peptide

*Sh. dysenteriae CMCC 51105* was purchased from the Nanjing Mao Jie Microorganism Technology Company Limited (Nanjing, China). *S. Typhi* and *S. aureus* were purchased from the Wenzhou Kangtai Bio-technology Company Limited (Wenzhou, Zhejiang, China). The bacterial strains were recovered by resuscitation fluid and cultured on an inclined surface for 24 h, and then they were transferred to the slant-culture medium. A small fraction was cultured in a liquid medium, and its absorbance at 625 nm was determined, which fell between 0.1–0.2 equivalents of half of the McFarland standard. Bacterial solution (1 mL) was added to the sterile culture dish, and then an appropriate amount of uncured solid medium was added and mixed with the bacterial fluid to be coagulated. The circular filter paper, which had been punched and sterilized, was soaked in the sample solution for a period of time. The papers were removed and placed in the culture dish and then cultured for 18–20 h at a 37 °C constant temperature to observe whether there was the formation of an inhibition zone. The formation of a bacteriostatic zone indicates that the sample has antibacterial activity, and the antibacterial activity can be qualitatively measured by the size of the bacteriostatic zone.

## 4. Conclusions

Cathelicidin-BF-30 has been successfully loaded in 4-arm-PEG-PLGA microspheres via double-emulsion solvent evaporation. Approximately 20% of this peptide was released from these microspheres during the first 12 h in vitro, and the delivery system released peptide over more than 15 days. Moreover, the peptides that were released from the microspheres presented high antibacterial activity against *Sh. dysenteriae CMCC 51105* and *S. typhi*. Furthermore, the secondary structure of BF-30 was not changed during the release period. Hence, the microspheres loaded with BF-30 achieved the aim of maintaining the sustained release and antimicrobial activity of BF-30. These prepared microspheres are a promising alternative method for cathelicidin-BF-30 delivery and even more consequential as a protective system to prevent peptide degradation.
